# Confinement‐Driven CO Spillover in CuAg@MSN Tandem Catalysts Boosts C_2_ Selectivity Toward Electrocatalytic CO_2_ Reduction

**DOI:** 10.1002/advs.76068

**Published:** 2026-06-11

**Authors:** Jiaying Zhang, Junjie Huang, Siying Zhang, Yanjia Cui, Chao Kong, Zijian Peng, Huihui Jiang, Sirui Deng, Zhuoyao Chen, Caili Yang, Ketong He, Zhen Li, Yibing Song, Gongwei Wang, Lin Zhuang

**Affiliations:** ^1^ College of Chemistry & Chemical Engineering and Key Laboratory for Preparation and Application of Ordered Structural Materials of Guangdong Province Shantou University Shantou China; ^2^ College of Petroleum and Chemical Engineering Longdong University Qingyang China; ^3^ College of Chemistry and Molecular Sciences Hubei Key Lab of Electrochemical Power Sources Wuhan University Wuhan China; ^4^ The Institute for Advanced Studies Wuhan University Wuhan China

**Keywords:** C_2_ product, CO_2_ reduction, confinement effect, In situ ATR‐SEIRAS, tandem catalyst

## Abstract

Tandem catalysts represent a promising paradigm for synergistic electrochemical CO_2_ reduction (CO_2_RR) to high‐value multi‐carbon (C_2_) products, yet coordinate regulation of key intermediates to boost C_2_ selectivity remains unclear. Herein, a dual‐phase templating coupled with gas‐phase reduction strategy is exploited to fabricate a series of CuAg@MSN tandem catalysts with tunable Cu/Ag mass ratios. A volcano‐type relationship between catalyst composition and C_2_ product selectivity is observed, with CuAg@MSN‐3 (15 wt.% Ag, 40 wt.% Cu) delivering the maximum C_2_ Faradaic efficiency (FE) of 75.4% at −1.4 V vs RHE. And a high FE of 63.5% and a large partial current density of 330 mA/cm^−2^ toward C_2_ products on CuAg@MSN‐3 at a cell voltage of 4.5 V in a membrane‐electrode assembly (MEA). In situ infrared spectroscopy and density functional theory (DFT) calculations jointly corroborate that CO generated on Ag sites spontaneously spills over and becomes confined within the mesopores of the MSN framework, markedly promoting CO re‐adsorption on adjacent Cu sites and elevating the ^*^CO surface coverage. This intermediate regulation effectively enhances the reaction rate of C─C coupling and consequently boosts C_2_ product selectivity. The present work exemplifies a rational, confinement‐guided design of tandem catalysts for steering reaction intermediates toward desired multicarbon products.

## Graphical abstract

The CuAg@MSN catalyst developed in this study promotes C─C coupling by regulating the coverage of ^*^CO intermediates through spatial confinement effects, significantly enhancing the selectivity of CO_2_ electroreduction for C_2_ product formation.

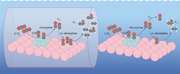

## 1 Introduction

The electrocatalytic reduction of carbon dioxide (CO_2_RR) represents a promising strategy toward sustainable carbon management. This approach can mitigate excessive CO_2_ emissions and alleviate associated environmental pressures, while converting CO_2_ into value‐added chemicals and fuels, thus closing the carbon cycle [1, 2]. Accordingly, CO_2_RR carries considerable significance for advancing sustainable development, supporting low‐carbon economies, and satisfying growing energy demands [3, 4] Of particular interest is the electrosynthesis of multi‐carbon (C_2_) products, including ethanol [5], ethylene [6], and n‐propanol [7], which offer high market value and widespread industrial applicability. A critical step during C_2_ generation is C─C bond formation, which is highly dependent on the surface coverage of ^*^CO intermediates and their adsorption strength on the catalyst surface [8, 9].

Copper uniquely provides moderate binding energy toward ^*^CO, enabling favorable C─C coupling between adjacent adsorbed intermediates [10, 11] For this reason, Cu remains the most effective monometallic catalyst for producing a wide range of C_2_ products from CO_2_. Nevertheless, conventional Cu catalysts often suffer from sluggish kinetics for the initial conversion of CO_2_ to CO [12, 13], leading to insufficient ^*^CO coverage. This bottleneck commonly results in unsatisfactory selectivity and activity toward C_2_ products. Improving the surface concentration of ^*^CO on Cu‐based catalysts therefore represents a central challenge in boosting C_2_ formation.

In recent years, tandem catalysis has emerged as a promising strategy to boost C_2_ production during CO_2_RR [14, 15, 16] By spatially separating distinct catalytic sites, individual elementary steps of CO_2_ reduction can proceed under optimized local environments, overcoming intrinsic constraints of single‐site catalysts. In typical designs, CO_2_ is first reduced efficiently to CO at sites such as noble metals [17, 18], transition metals [19, 20], or metal phthalocyanines [21, 22] The in situ‐generated CO can then re‐adsorb onto Cu sites, enriching ^*^CO coverage and promoting C─C coupling to form C_2_ products. This strategy generally improves C_2_ selectivity and overall reaction efficiency. However, a considerable fraction of the produced CO diffuses into the bulk electrolyte before re‐adsorption, which limits further performance enhancement [23].

Nanoporous catalysts offer good structural stability and a semi‐enclosed microenvironment that can confine reaction intermediates [24, 25, 26, 27, 28]. Such spatial confinement suppresses the escape of CO molecules, reduces CO loss, and elevates the local CO concentration near Cu surfaces. This favors the formation of densely adsorbed ^*^CO intermediates and promotes C─C dimerization. In our previous study, we developed a yolk–shell Cu@HCS catalyst [29] The unique yolk–shell structure effectively hindered CO diffusion and enhanced re‐adsorption, leading to high ^*^CO coverage on the Cu surface. The improved ^*^CO population then promoted C─C coupling and boosted C_2_ selectivity. The optimized Cu@HCS catalyst achieved a C_2_ Faradaic efficiency of 69.7% at −1.4 V vs. RHE. These results suggest that integrating tandem catalysis with spatial confinement effects offers a viable route to further enhance C_2_ selectivity.

In this work, we fabricated a Cu‐Ag tandem catalyst embedded in mesoporous self‐polymerized dopamine nanospheres (denoted as MSN) using a dual‐phase template method followed by gas‐phase reduction. The catalyst exhibits outstanding selectivity for C_2_ products during CO_2_ electroreduction. Under comparable metal loadings, CuAg@MSN outperforms the carbon‐supported control (CuAg/xc‐72) significantly, the C_2_ Faradaic efficiency rises from 30.1% to 75.4% at −1.4 V vs. RHE. Structural characterization and density functional theory calculations reveal that the mesoporous MSN matrix confines ^*^CO intermediates, slowing their diffusion and enhancing re‐adsorption at Cu sites. The increased ^*^CO coverage reduces the energy barrier for C─C coupling, thereby promoting efficient C_2_ production. This study demonstrates a general strategy to regulate intermediate behavior via support‐induced confinement, providing a rational design principle for improving both activity and selectivity in CO_2_ electroreduction toward multi‐carbon products.

## 2 Experimental Section

### 2.1 Chemicals and Materials

Copper nitrate hydrate (Cu(NO_3_)_2_·3H_2_O, 99%) and silver nitrate (AgNO_3_, 99%) were obtained from Aladdin Biochemical Technology Co., Ltd. Dopamine hydrochloride (DA, 99%) and 1,3,5‐trimethylbenzene (TMB, 98%) were supplied by Saen Chemical Technology Co., Ltd. Pluronic F‐127 (F127, 99%) was purchased from Sigma–Aldrich. Potassium bicarbonate (KHCO_3_, 99.5%) was acquired from Macklin Biochemical Co., Ltd. Vulcan XC‐72 carbon black was provided by SCI Materials. Anhydrous ethanol (99.99%) was purchased from Xilong Scientific Co., Ltd. All chemicals were used as received without further purification. Throughout the synthesis of catalysts and related materials in this work, deionized water with a resistivity of 18.2 MΩ·cm was used exclusively.

### 2.2 Synthesis of MSN

The synthesis of MSN is based on previously reported methods [30] and can be summarized as follows: In a 250 mL round‐bottom flask, 50 mL of ethanol and 50 mL of water were added successively, and the flask was sealed with a glass stopper. The flask was then placed on a magnetic stirrer and secured with a base. A magnetic stirring bar was introduced, and the mixture was stirred at a speed of 300 r/min for 1 min. Subsequently, 1.0 g of the triblock copolymer Pluronic F127 was accurately weighed and added to the ethanol/water mixture, followed by continuous stirring for 15 min to ensure the copolymer was fully dispersed. An appropriate amount of TMB solution was added to the mixture using a pipette, and the stirring was continued for another 15 min to ensure thorough mixing of all chemicals and solutions, resulting in the formation of the F127/TMB monoparticle system. Next, 750 mg of dopamine hydrochloride (DA) was precisely weighed and added to the system, and the mixture was stirred for 10 min to achieve a well‐mixed F127/TMB/DA composite solution. Subsequently, ammonia solution was added to the system and stirred to induce the polymerization of DA and guide the assembly of monoparticles into mesoporous polydopamine (mesoPDA) material. After 12 h of reaction, the mixture was washed with ethanol until the supernatant became clear and transparent. The centrifuged precipitate was then collected and dried. Finally, the product was calcined in an Ar atmosphere at 800°C for 2 h to obtain MSN.

### 2.3 Synthesis of CuAg@MSN

100 mg of MSN was placed into a 50 mL round‐bottom flask, followed by the addition of 15 mL deionized water. Under stirring conditions, stoichiometric Cu(NO_3_)_2_·3H_2_O and AgNO_3_ were sequentially added to the mixture. Subsequently, the mixture was subjected to ultrasonication for 10 min to ensure uniform dispersion. After ultrasonication, the mixture was continuously stirred at a rate of 400 r/min for 24 h. Upon completion of the reaction, the mixture was transferred to an oven and dried at 80°C for 12 h to remove moisture. Finally, the dried sample was placed in a tube furnace and subjected to a reduction process under a mixed atmosphere of hydrogen and argon. The sample was heated to 350°C at a rate of 5°C/min and maintained at this temperature for 2 h. Through the above process, the final material was successfully synthesized, denoted as CuAg@MSN‐x, where x corresponds to the loading of Ag NPs (x = 1 for 5%, x = 2 for 10%, x = 3 for 15%, x = 4 for 20%, and x = 5 for 25%).

### 2.4 Synthesis of Cu@MSN, Ag@MSN and CuAg/xc‐72

The Cu@MSN, Ag@MSN, and CuAg/xc‐72 catalysts were synthesized following the same procedure as the CuAg@MSN‐3 catalyst.

Additionally, details of material characterizations, electrochemical measurements, electrochemically active surface area (ECSA) determinations, in situ attenuated total reflection surface‐enhanced infrared absorption spectroscopy (ATR‐SEIRAS) measurements, and density functional theory (DFT) calculations are provided in the Supporting Information.

## 3 Results and Discussion

### 3.1 Material Characterizations

The morphology and nanostructure of MSN were examined by scanning electron microscopy (SEM) and high‐resolution transmission electron microscopy (TEM). As shown in Figure S1a,b, the as‐prepared MSN possesses a uniform spherical morphology with a narrow particle size distribution centered at around 210 nm. Ordered mesoporous structures can be clearly observed across the entire spherical framework. N_2_ adsorption–desorption isotherms were employed to characterize the surface area and pore structure of MSN (Figure S2 and Table S1). The sample exhibits notable porosity, with a specific surface area of 228.71 m^2^/g, a total pore volume of 0.073 cm^3^/g, and an average pore diameter of 5.5 nm. X‐ray diffraction (XRD) patterns display no obvious crystalline diffraction peaks (Figure S3), verifying the amorphous structure of MSN. Such an amorphous mesoporous architecture favors the diffusion and adsorption of metal ions from the precursor solution [31] It also acts as a suitable support for the uniform dispersion and in situ reduction of metal species during thermal treatment, which is beneficial for the formation of highly dispersed metal‐based composite catalysts.

**FIGURE 1:**
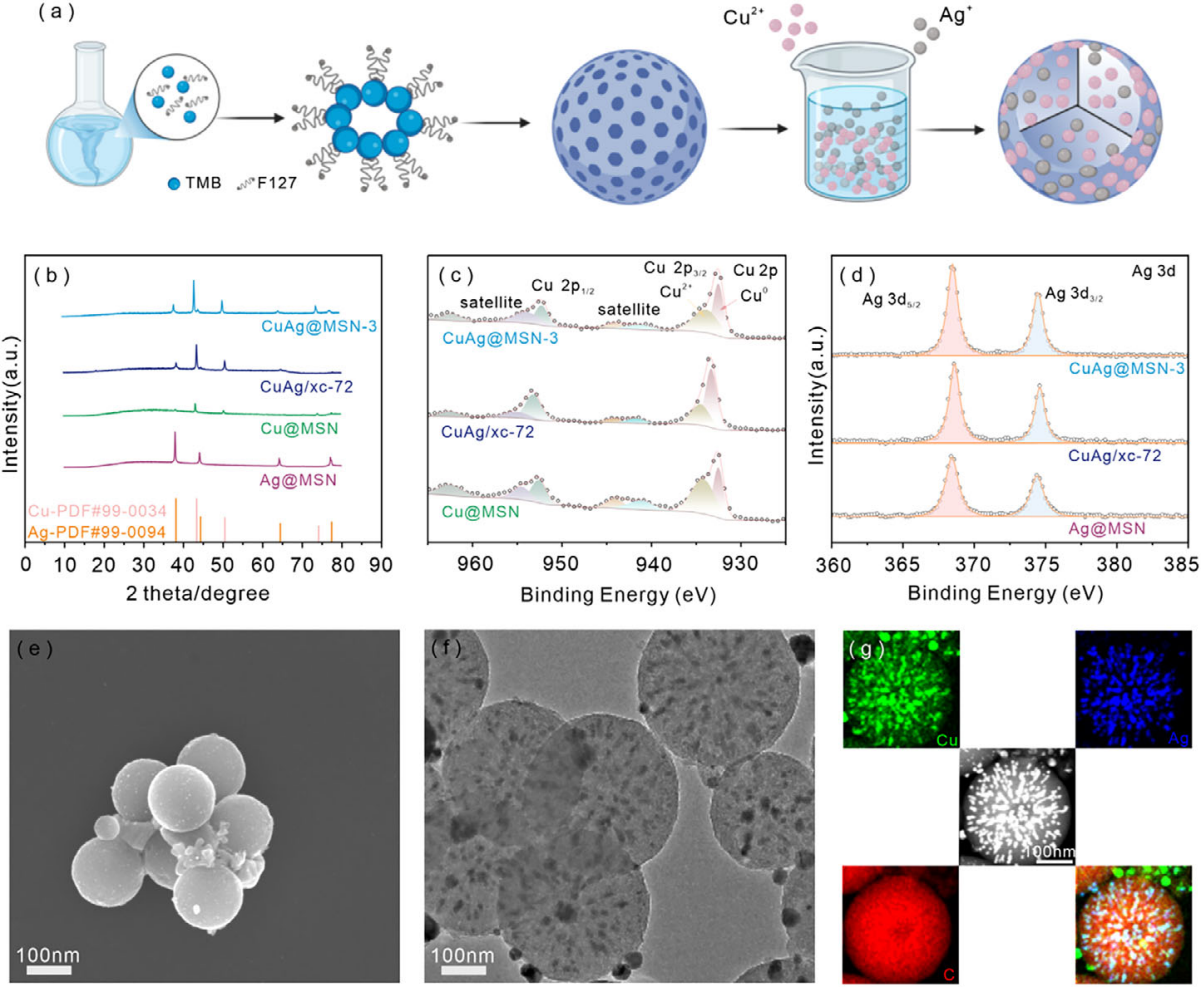
(a) The scheme for synthesizing the CuAg@MSN catalyst. (b) The XRD patterns of the CuAg@MSN,CuAg/xc‐72,Cu@MSN and Ag@MSN samples. (c) The XPS spectra of the Cu specie in CuAg@MSN,CuAg/xc‐72 and Cu@MSN samples. (d) The XPS spectra of the Ag specie in CuAg@MSN, CuAg/xc‐72, and Ag@MSN samples. (e) The SEM image of the CuAg@MSN sample. (f) The TEM image of the CuAg@MSN sample. (g) Corresponding elemental mapping of Cu, C, and Ag species in the CuAg@MSN sample.

A series of CuAg@MSN catalysts were prepared via a dual‐phase templating approach combined with gas‐phase reduction, as schematically illustrated in Figure 1a. To systematically clarify their structural properties, x‐ray diffraction (XRD) measurements were performed. As shown in Figure S4, all CuAg@MSN samples exhibit consistent diffraction patterns, with characteristic peaks at 43.3°, 50.4°, and 74.1° corresponding to the (111), (200), and (220) crystal planes of face‐centered cubic (fcc) metallic Cu (PDF#99‐0034). Meanwhile, distinct diffraction peaks at 38.2°, 44.5°, 64.5°, and 77.5° are clearly attributed to the (111), (200), (220), and (311) planes of fcc metallic Ag (PDF#99‐0094). No obvious peak shifts or additional peaks corresponding to metal oxides or alloy phases were detected, confirming the successful incorporation of both metallic Cu and Ag into the MSN framework.

Notably, the intensity of Ag‐related diffraction peaks gradually increased with increasing AgNO_3_ precursor concentration, indicating that the composition of CuAg@MSN catalysts can be precisely regulated by adjusting the precursor dosage. For comparison, the structural properties of Cu@MSN, Ag@MSN, and CuAg/xc‐72 catalysts wereA also characterized by XRD (Figure 1b). The CuAg/xc‐72 catalyst exhibited the same characteristic diffraction peaks for metallic Cu and Ag as CuAg@MSN, demonstrating that both Cu and Ag exist in the metallic state in these bimetallic catalysts. In contrast, the monometallic Cu@MSN and Ag@MSN catalysts only showed the standard diffraction peaks of metallic Cu and metallic Ag, respectively. These results confirm the successful synthesis of all three reference catalyst systems.

The chemical states of Cu@MSN, Ag@MSN, CuAg/xc‐72, and CuAg@MSN catalysts were further examined by x‐ray photoelectron spectroscopy (XPS). As presented in Figure 1c, the high‐resolution Cu 2p spectra of Cu@MSN, CuAg/xc‐72, and CuAg@MSN show two prominent peaks at 932.5 and 952.3 eV, which can be assigned to the 2p_3/2_ and 2p_1/2_ signals of Cu^+^/Cu^0^, respectively. The Cu LMM Auger spectrum (Figure S5) further verifies the presence of Cu^0^, with a characteristic peak at around 572.5 eV, matching well with metallic Cu. These results indicate that Cu in these three catalysts mainly exists in the metallic form. Weak peaks at 933.5 and 953.3 eV are also visible, corresponding to Cu^2+^ from CuO, which is likely caused by surface oxidation upon exposure to air [32] No obvious binding energy shifts are observed among the three samples, suggesting that the chemical state of Cu is largely unchanged. The Ag 3d spectra of Ag@MSN, CuAg/xc‐72, and CuAg@MSN are displayed in Figure 1d. Two characteristic peaks appear at 368.5 and 374.3 eV, matching well with metallic Ag 3d_5/2_ and 3d_3/2_. The nearly identical binding energies across these catalysts confirm that Ag is present in its metallic state without obvious chemical variation. Overall, the XPS results reveal that Cu and Ag in both CuAg/xc‐72 and CuAg@MSN exist as metallic species, with no obvious shifts or new peaks typical of alloy or intermetallic compound formation. This indicates that the two metals are present in a bimetallic configuration without strong electronic interaction.

The morphological features and structural integrity of Cu@MSN, Ag@MSN, CuAg/xc‐72, and CuAg@MSN were investigated using SEM and TEM. SEM images (Figure 1e) confirm that CuAg@MSN retains the uniform spherical morphology of the parent MSN framework, indicating that the mesoporous structure is preserved during the thermal reduction process. A small number of nanoparticles are visible on the external surface, which we attribute to incomplete infiltration of the metal precursors into the mesoporous channels. This structural integrity is general to the series, as Cu@MSN and Ag@MSN exhibit analogous morphologies (Figure S6a,b). For comparison, CuAg/xc‐72 displays the typical aggregated morphology of carbon black supports, where individual metal nanoparticles are not readily distinguishable (Figure S6c). TEM was used to resolve the dispersion of metal nanoparticles at the nanoscale. As shown in Figure 1f, Cu and Ag nanoparticles are uniformly dispersed within the mesoporous channels of CuAg@MSN. This confined distribution is also observed for the monometallic Cu@MSN and Ag@MSN catalysts (Figure S7a,b). In stark contrast, CuAg/xc‐72 shows metal nanoparticles deposited on the external surface of the carbon support (Figure S7c). High‐resolution TEM imaging (Figure S8) reveals clear lattice fringes with d‐spacings of 0.22 and 0.21 nm, corresponding to the Cu(111) and Ag(111) planes, respectively. And the corresponding Fourier transform patterns clearly show that the reciprocal lattice points with 1/d = 4.48 nm^−1^ and 1/d = 4.23 nm^−1^ correspond to the Cu(111) and Ag(111) facets, respectively. This confirms the metallic state of both elements and rules out the formation of amorphous phases.

Elemental mapping via energy‐dispersive x‐ray spectroscopy (EDS) further corroborates these findings (Figure 1g and Figure S9). While Cu and Ag are homogeneously distributed across all samples, the elemental maps of the bimetallic catalysts show minimal overlap between the Cu and Ag signals. This distinct spatial separation confirms that Cu and Ag exist as discrete nanoparticles rather than forming a homogeneous alloy or intermetallic compound within the CuAg@MSN architecture. N_2_ adsorption–desorption isotherms were used to characterize the specific surface area, pore volume, and pore size distribution of Cu@MSN, Ag@MSN, CuAg/xc‐72, and CuAg@MSN catalysts (Figure S10). Under identical relative pressure conditions, Cu@MSN, Ag@MSN, and CuAg@MSN exhibit much higher nitrogen adsorption capacities than CuAg/xc‐72, indicating significantly larger specific surface areas for the former three samples. BET calculations (Table S2) quantify these differences: Cu@MSN, Ag@MSN, and CuAg@MSN have specific surface areas of 266.56, 219.56, and 188.51 m^2^/g, respectively, far exceeding the 74.92 m^2^/g of CuAg/xc‐72. Distinct hysteresis loops are observed for Cu@MSN, Ag@MSN, and CuAg@MSN, whereas no such loop is present for CuAg/xc‐72. This indicates that the MSN‐supported catalysts possess abundant, uniformly distributed pore structures, in contrast to the carbon‐supported counterpart. BJH analysis further confirms narrow pore size distributions for the MSN‐supported catalysts (Figure S11 and Table S2), with average pore diameters of 5.4, 8.3, and 8.0 nm for Cu@MSN, Ag@MSN, and CuAg@MSN, respectively. In contrast, CuAg/xc‐72 exhibits a broad pore size distribution and is nearly nonporous, a characteristic feature of the Vulcan xc‐72 carbon support. Additionally, the pore volumes of Cu@MSN, Ag@MSN, and CuAg@MSN are 0.068, 0.061, and 0.063 cm^3^/g, respectively, slightly lower than the 0.073 cm^3^/g of the pristine MSN framework. This confirms preferential encapsulation of metal nanoparticles within the MSN mesoporous channels. Such hierarchical porosity is critical for electrocatalytic CO_2_RR performance, as it facilitates efficient mass transfer of H_2_O and CO_2_ to active sites while optimizing the confinement of reaction intermediates.

### 3.2 Electrocatalytic CO_2_RR Performance

The electrocatalytic CO_2_ reduction performance was evaluated in a dual‐chamber H‐type cell separated by a Nafion 117 proton‐exchange membrane. Gaseous products were quantified by online gas chromatography, and liquid products were characterized using ^1^H NMR spectroscopy. Prior to systematic mechanistic investigations, we systematically optimized the MSN pore size and Cu/Ag bimetallic ratio, with the corresponding results displayed in Figures S12–S15. The results reveal a strong nonlinear correlation between C_2_ product selectivity and the structural/compositional parameters, including MSN pore size and metal loading ratio. Among all prepared catalysts, the CuAg@MSN‐3 catalyst (MSN pore size: 9.5 nm, Cu loading: 40%, Ag loading: 15%) exhibits the optimal electrocatalytic performance, delivering a maximum C_2_ Faradaic efficiency of 75.4%. Accordingly, CuAg@MSN‐3 was chosen as the optimal catalyst for further studies, aiming to reveal the synergistic role of Cu‐Ag bimetallic sites and the confinement effect imposed by the mesoporous MSN support toward boosted C_2_ selectivity in electrocatalytic CO_2_RR.

To highlight the advantages of CuAg@MSN‐3 for CO_2_ to C_2_ conversion, the electrocatalytic CO_2_RR performance of CuAg@MSN‐3, Cu@MSN, Ag@MSN, and CuAg/xc‐72 was evaluated in 0.1 m KHCO_3_ electrolyte. LSV curves of these catalysts are presented in Figure S16. At the same applied potential, CuAg@MSN‐3 exhibits a significantly higher current density than Cu@MSN, Ag@MSN, and CuAg/xc‐72, indicating excellent electrocatalytic activity toward CO_2_RR. The corresponding product distributions are shown in Figure S17a–d. Obvious differences are observed in the Faradaic efficiencies for H_2_, CH_4_, CO, C_2_H_4_, C_2_H_5_OH, and HCOOH over the four catalysts. CuAg@MSN‐3 favors the formation of C_2_ products, whereas CuAg/xc‐72 and Cu@MSN mainly produce H_2_, and Ag@MSN generates CO as the dominant product. These results demonstrate that the combination of Cu‐Ag bimetallic sites and the confinement effect of the MSN support effectively promotes the selective production of C_2_ products in CO_2_ electroreduction.

**FIGURE 2:**
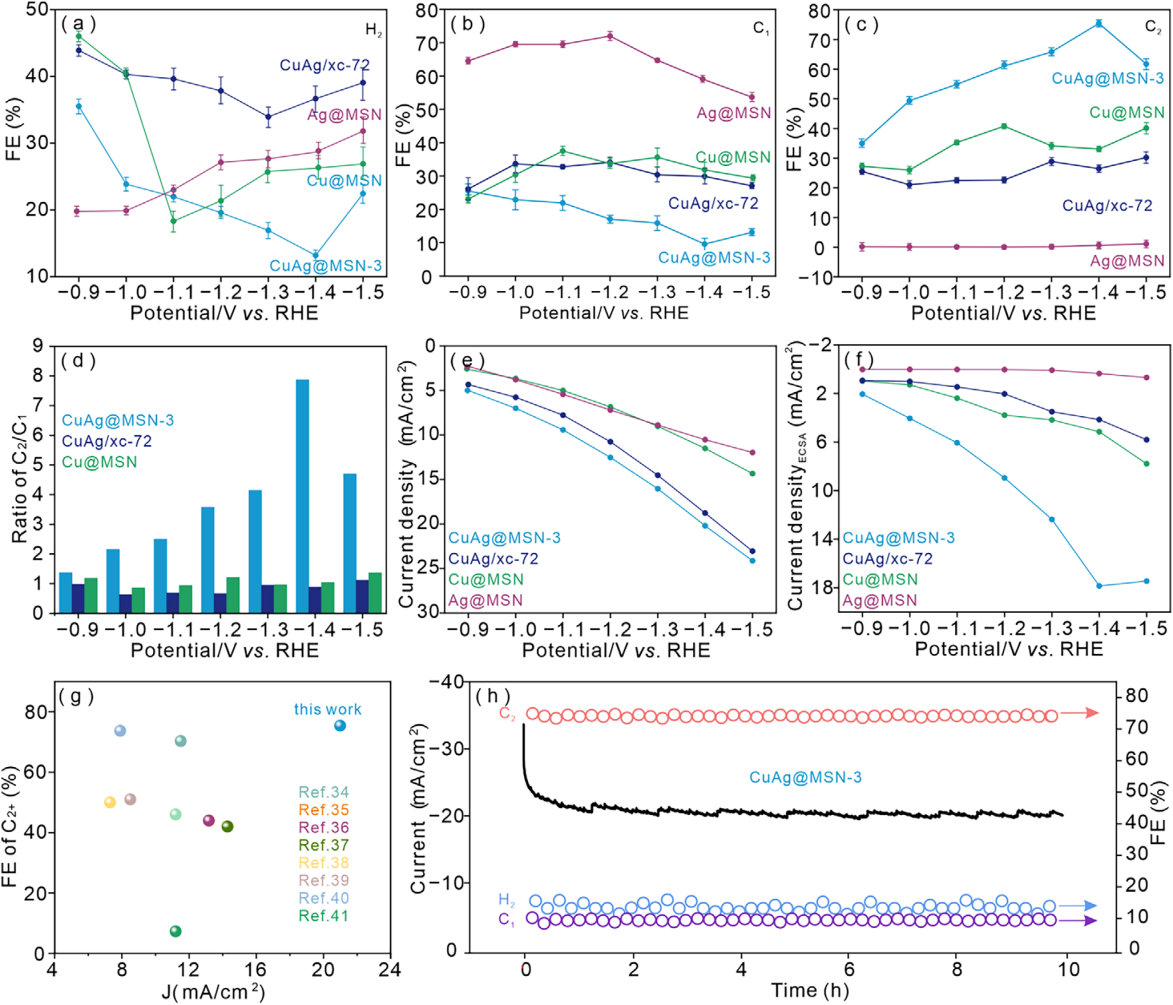
(a) The FE of H_2_ products on CuAg@MSN‐3, Cu@MSN, Ag@MSN and CuAg/xc‐72 catalysts. (b) The FE of C_1_ products on CuAg@MSN‐3, Cu@MSN, Ag@MSN and CuAg/xc‐72 catalysts. (c) The FE of C_2_ products on CuAg@MSN‐3, Cu@MSN, Ag@MSN and CuAg/xc‐72 catalysts. (d) The ratio of C_2_/C_1_ products on CuAg@MSN‐3, Cu@MSN and CuAg/xc‐72 catalysts. (e) The current density of C_1_ and C_2_ products on CuAg@MSN‐3, Cu@MSN,Ag@MSN and CuAg/xc‐72 catalysts. (f) The current density_ECSA_of C_1_ and C_2_ products on CuAg@MSN‐3, Cu@MSN, Ag@MSN and Cu/xc‐72 catalysts. (g) Comparison of the CuAg@MSN‐3 with the reported state‐of‐the‐art Cu‐based catalysts. (h) Long‐term catalysis stability of CuAg@MSN‐3.

The potential‐dependent Faradaic efficiencies of H_2_, C_1_, and C_2_ products over CuAg@MSN‐3, CuAg/xc‐72, Cu@MSN, and Ag@MSN are compared in Figure 2a–c. The H_2_ FE of the MSN‐supported catalysts (CuAg@MSN‐3, Cu@MSN, and Ag@MSN) is consistently lower than that of CuAg/xc‐72 across all potentials (Figure 2a), demonstrating that the porous MSN framework inherently suppresses the hydrogen evolution reaction (HER). For CuAg@MSN‐3 specifically, the H_2_ FE drops sharply between −0.9 and −1.4 V vs. RHE. This trend suggests that hydrogen‐related intermediates on the catalyst surface are preferentially involved in the hydrogenation of CO_2_ or its derivatives, rather than undergoing H–H coupling to form H_2_. Figure 2b highlights the strong selectivity of Ag@MSN for C_1_ products (primarily CO), consistent with the known catalytic behavior of Ag [33]. In the bimetallic CuAg@MSN‐3 catalyst, the CO generated at Ag sites elevates the local CO concentration at the adjacent Cu sites. This enrichment increases the surface coverage of ^*^CO intermediates and facilitates C─C coupling, leading to a remarkable C_2_ FE of 75.4% (Figure 2c), a 2.3‐fold improvement over the monometallic Cu@MSN catalyst. The superior performance over CuAg/xc‐72 further underscores the critical role of the MSN framework's confinement effect in boosting C_2_ selectivity.

The C_2_/C_1_ FE ratio, a key metric for evaluating C─C coupling efficiency, is plotted in Figure 2d. For CuAg@MSN‐3, this ratio follows a volcano‐type trend with increasing potential, peaking at 7.9 at −1.4 V vs. RHE. This value significantly outperforms those of CuAg/xc‐72 and Cu@MSN. Together, these results confirm that the synergistic effect of CuAg bimetallic active sites and mesoporous confinement can effectively regulate the product selectivity of CO_2_ reduction, achieving a significant enhancement in C_2_ product.

Current density is a critical parameter for assessing electrocatalytic CO_2_RR performance. As shown in Figure 2e, all four catalysts (CuAg@MSN‐3, CuAg/xc‐72, Cu@MSN, and Ag@MSN) exhibit increasing geometric current densities with more negative polarization potentials. Notably, CuAg@MSN‐3 maintains consistently higher current densities across the entire potential range, indicating superior intrinsic activity toward CO_2_RR. To clarify the origin of this activity advantage, we measured the electrochemically active surface area (ECSA) of each catalyst via double‐layer capacitance measurements (Figure S18 and Table S3). The ECSA values follow the order, CuAg/xc‐72 (94 cm^2^/mg) > CuAg@MSN‐3 (67 cm^2^/mg) > Cu@MSN (58 cm^2^/mg) > Ag@MSN (15 cm^2^/mg). Despite having only 71.2% of the ECSA of CuAg/xc‐72, CuAg@MSN‐3 exhibits substantially higher ECSA‐normalized current density (Figure 2f). Most notably, it achieves a C_2_ partial current density of 12.4 mA/cm^2^, which is significantly higher than those of the reference catalysts. These results confirm that while ECSA contributes to catalytic performance, the intrinsic properties of the active sites are the decisive factor. The superior activity of CuAg@MSN‐3 stems from the synergistic interplay between Cu‐Ag bimetallic sites and mesoporous confinement, which optimizes ^*^CO intermediate adsorption and facilitates C─C coupling. As illustrated in Figure 2g, CuAg@MSN‐3 achieves a high C_2_ selectivity of 75.4% at a current density of 22 mA/cm^2^, outperforming many previously reported Cu‐based catalysts for CO_2_ to C_2_ conversion [34, 35, 36, 37, 38, 39, 40, 41].

The stability of CuAg@MSN‐3, CuAg/xc‐72, Cu@MSN, and Ag@MSN catalysts was evaluated under CO_2_ electroreduction conditions (Figure 2h and Figure S19). All catalysts maintained stable product selectivity over 10 h of continuous electrolysis at −1.4 V vs. RHE, with Faradaic efficiency fluctuations of less than 5% for H_2_, C_1_, and C_2_ products. Notably, CuAg@MSN‐3, Cu@MSN, and Ag@MSN exhibited excellent current density stability throughout the test. In contrast, CuAg/xc‐72 suffered significant activity decay, with its current density dropping from 19.1 to 16.9 mA/cm^2^. Post‐electrolysis characterization revealed distinct structural changes among the catalysts. SEM and TEM images (Figures S20 and S21) showed well‐preserved nanoparticle morphologies for the MSN‐supported catalysts (CuAg@MSN‐3, Cu@MSN, Ag@MSN), whereas CuAg/xc‐72 displayed obvious nanoparticle aggregation. Quantitative XRD analysis, combined with Scherrer equation calculations (Figure S22 and Table S4), further confirmed these observations. The crystallite sizes of CuAg@MSN‐3, Cu@MSN, and Ag@MSN remained nearly unchanged after long‐term electrolysis, while the Cu in CuAg/xc‐72 exhibited a substantial increase in crystallite size (>30% growth). These consistent results across multiple characterization techniques demonstrate that the MSN framework effectively suppresses nanoparticle reconstruction through physical confinement. The exceptional stability of MSN‐supported catalysts highlights the potential of MSN as a superior support for designing durable CO_2_ electroreduction catalysts.

### 3.3 Mechanism of CuAg@MSN‐3 Catalyst for CO_2_RR

**FIGURE 3:**
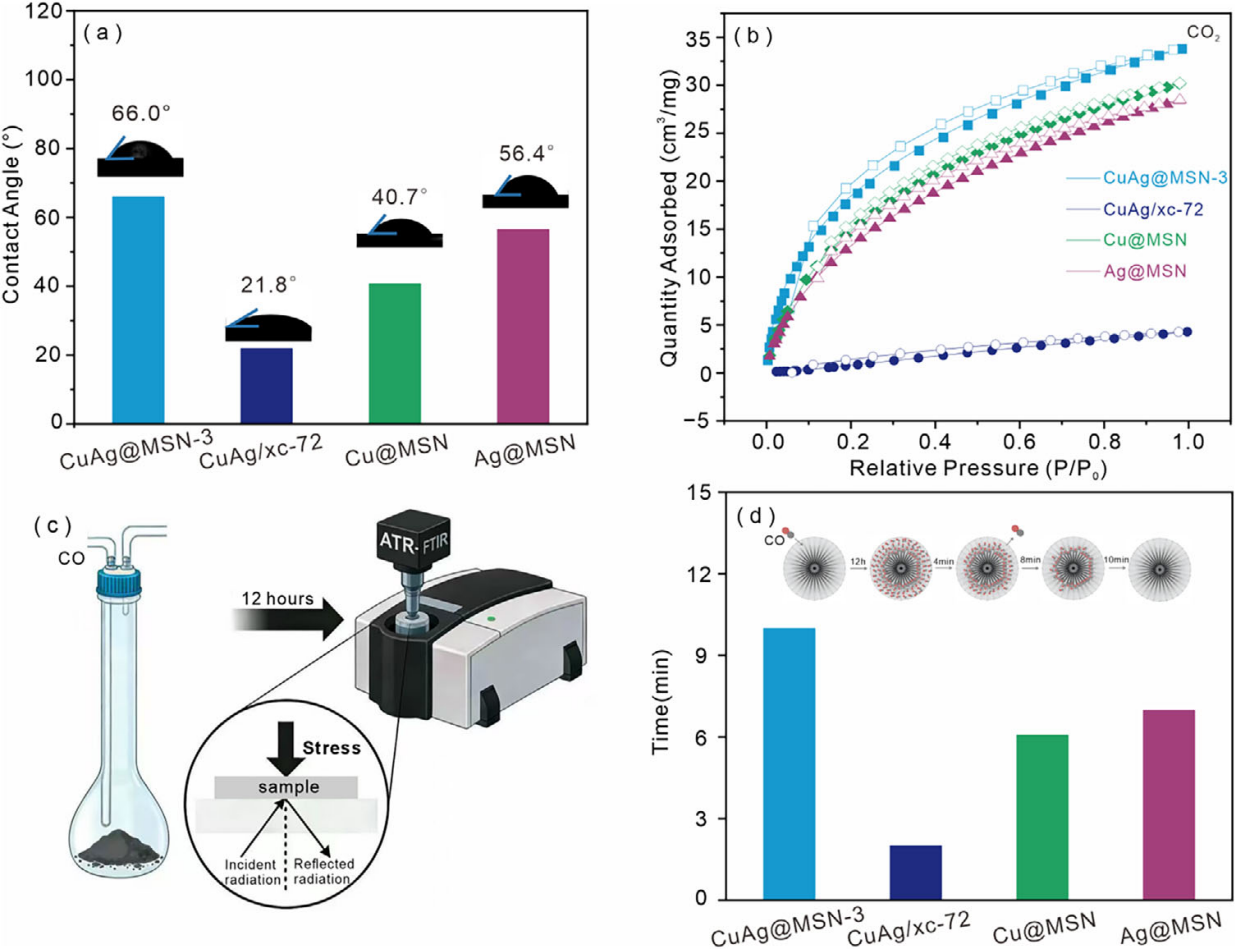
(a) Contact angle of data CuAg@MSN‐3, Cu@MSN, Ag@MSN, and CuAg/xc‐72 catalysts. (b) Carbon dioxide sorption and pore textural properties of CuAg@MSN‐3, Cu@MSN, Ag@MSN and CuAg/xc‐72 catalysts. (c) Schematic illustration of ATR‐FTIR tests. (d) The retention times of CO in CuAg@MSN‐3, Cu@MSN, Ag@MSN and CuAg/xc‐72 catalysts. Prior to characterization, catalysts were pretreated in Ar and CO atmospheres for 12 h. All tests were conducted under ambient conditions.

To understand how CuAg@MSN‐3 suppresses the HER during CO_2_ electroreduction, we evaluated the wettability of all catalysts via static contact angle measurements (Figure 3a). The contact angles of CuAg@MSN‐3, Ag@MSN, Cu@MSN, and CuAg/xc‐72 are 66.0°, 56.4°, 40.7°, and 21.8°, respectively. These values clearly show that the MSN‐supported catalysts (CuAg@MSN‐3, Ag@MSN, Cu@MSN) are significantly more hydrophobic than CuAg/xc‐72, demonstrating that the porous MSN framework effectively enhances catalyst surface hydrophobicity. This increased hydrophobicity is hypothesized to reduce the concentration of H_2_O molecules at the catalyst–electrolyte interface, thereby suppressing HER activity [42]. Given the typical correlation between hydrophobicity and gas affinity, we further assessed the CO_2_ adsorption capacity of the catalysts via CO_2_ adsorption measurements at 0°C (Figure 3b). Under identical relative pressure conditions, the CO_2_ adsorption capacity increases in the order: CuAg/xc‐72 < Ag@MSN < Cu@MSN < CuAg@MSN‐3. At p/p_0_ = 1, the CO_2_ adsorption capacities are 4.293, 28.489, 30.221, and 33.805 cm^3^/mg for these catalysts, respectively. These results confirm that metal‐loaded MSN catalysts exhibit substantially higher CO_2_ adsorption capacities than the carbon‐supported counterpart. A high local CO_2_ concentration at the catalyst surface favors faster CO_2_ reduction kinetics, thereby promoting the overall CO_2_RR process.

To verify this mechanism, Tafel slope analysis was carried out (Figure S23). In a N_2_ atmosphere, the Tafel slopes reveal that CuAg/xc‑72 displays the fastest HER kinetics, followed by Cu@MSN, Ag@MSN, and CuAg@MSN‑3. This trend agrees well with the contact angle measurements. In a CO_2_ atmosphere, the Tafel slopes are 102, 151, 158, and 162 mV dec^−1^ for CuAg@MSN‑3, Ag@MSN, Cu@MSN, and CuAg/xc‑72, respectively. These results demonstrate that CuAg@MSN‑3 exhibits the most rapid CO_2_ reduction kinetics, further confirming its excellent CO_2_RR performance.

To further clarify the role of the porous MSN framework in regulating CO diffusion during CO_2_ reduction, we used attenuated total reflection Fourier transform infrared spectroscopy (ATR‐FTIR) to characterize the catalysts (Figure 3c). For each catalyst, two aliquots were prepared and pretreated with Ar and CO gas for 12 h to remove residual air and ensure the reliability of the ATR‐FTIR measurements. ATR‐FTIR spectra of Ar‐pretreated samples served as background references (Figure S24). A characteristic absorption band in the range of 2145–2211 cm^−1^ was observed, corresponding to the vibrational mode of gaseous CO [43]. As the reaction proceeded, the intensity of this band gradually decreased and eventually vanished, indicating that CO molecules were desorbing from the catalyst surface. The CO retention times for different catalysts are summarized in Figure 3d: CuAg@MSN‐3, Ag@MSN, and Cu@MSN exhibit retention times of 10, 6, and 7 min, respectively, significantly longer than the 2 min observed for CuAg/xc‐72. This confirms that the porous MSN framework effectively retards CO diffusion, reducing CO desorption from the catalyst surface. This confinement effect promotes CO re‐adsorption at Cu active sites, increases ^*^CO intermediate coverage on the Cu surface, and thereby accelerates C─C coupling. This explains why CuAg@MSN‐3 achieves the highest C_2_ product selectivity.

**FIGURE 4:**
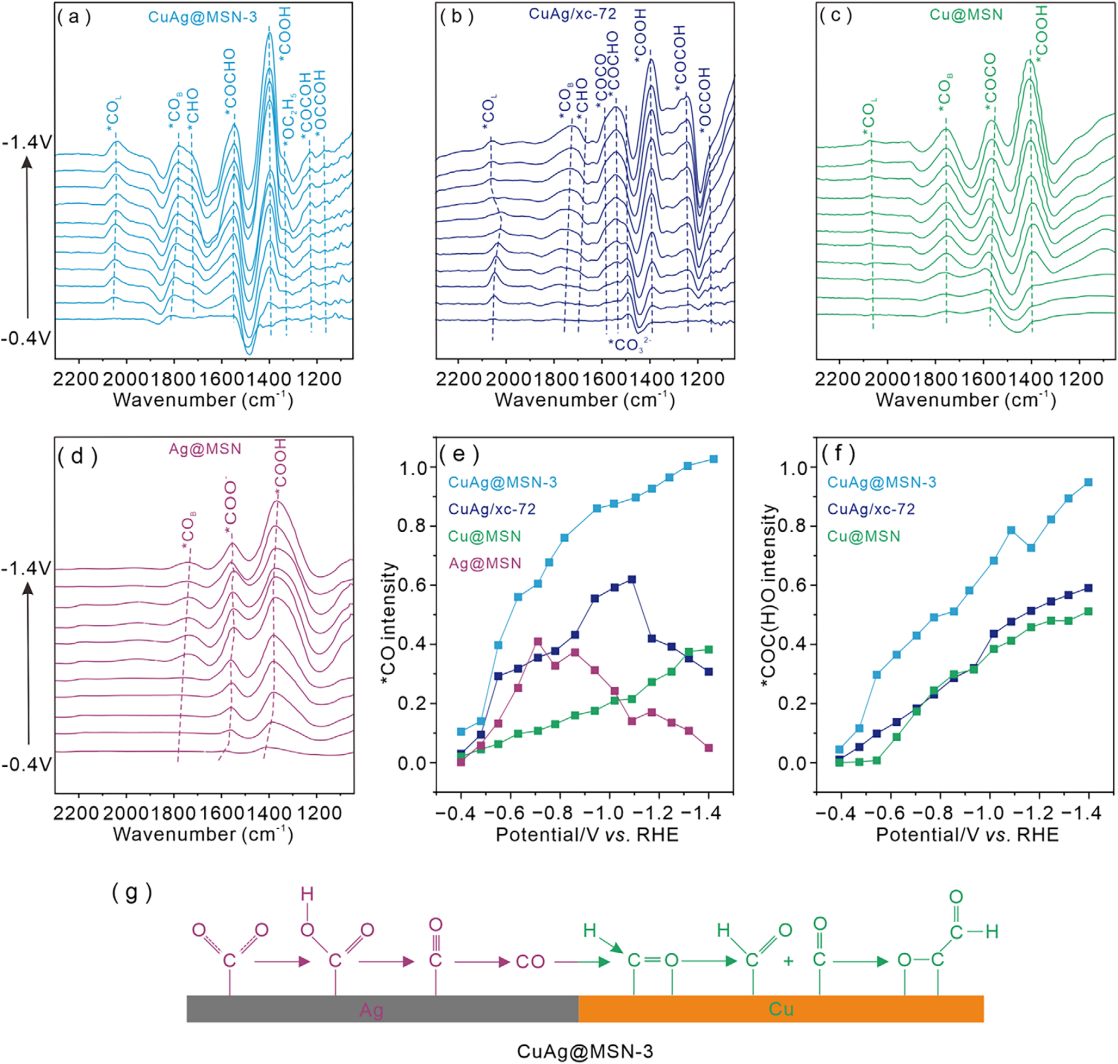
The ATR‐SEIRAS spectra recorded in CO_2_‐saturated 0.5 m KHCO_3_ solution during the CO_2_RR on (a) CuAg@MSN‐3; (b) CuAg/xc‐72; (c) Cu@MSN and (d) Ag@MSN. Peak intensities of normalized (e) ^*^CO and (f) ^*^COCHO + ^*^COCO on CuAg@MSN‐3, CuAg/xc‐72, Cu@MSN and Ag@MSN catalysts. (g) The schematic illustration of CO_2_ electroreduction over the CuAg@MSN‐3 catalyst.

To gain further insight into the mechanism by which CuAg@MSN‑3 catalyzes CO_2_ conversion to C_2_ products, in‑situ ATR‑SEIRAS was used to monitor key reaction intermediates. Tests were performed in CO_2_‑saturated 0.5 M KHCO_3_ solution, with spectra collected from –0.4 to –1.4 V vs. RHE. The spectrum recorded at –0.4 V was used as the background reference (Figure 4a–d). For all catalysts (CuAg@MSN‑3, CuAg/xc‑72, Cu@MSN, and Ag@MSN), a band assigned to the ^*^COOH intermediate appeared at approximately 1400 cm^−1^, identifying this species as a critical intermediate for CO formation [44, 45]. Compared with CuAg@MSN‑3, CuAg/xc‑72, and Cu@MSN, the ^*^COOH peak of Ag@MSN showed an obvious potential‑dependent shift. This shift can be attributed to strong chemical adsorption between ^*^COOH and the Ag surface, which weakens the C─O bond [46, 47]. These results indicate that Ag sites effectively adsorb and activate CO_2_ to form ^*^COOH, consistent with the high intrinsic CO_2_RR activity of the Ag component.

Bands in the 2000–2200 cm^−1^ region are assigned to the stretching vibration of linearly adsorbed CO (^*^CO_L_). Notably, no obvious peak was observed for Ag@MSN, which is likely due to the weak adsorption of ^*^CO_L_ on Ag, leading to facile desorption under reaction conditions [48, 49], In contrast, for both CuAg@MSN‑3 and CuAg/xc‑72, CO produced at Ag sites can migrate to Cu surfaces and re‑adsorb, thereby increasing the coverage of ^*^CO intermediates. Furthermore, the porous MSN framework imposes a confinement effect that restricts the escape of CO into the electrolyte, further promoting CO re‑adsorption on Cu. As a result, CuAg@MSN‑3 exhibits the highest ^*^CO coverage among all catalysts. Figure 4e summarizes the ^*^CO coverage, revealing the following trend, CuAg@MSN‑3 > CuAg/xc‑72 > Cu@MSN > Ag@MSN. The elevated coverage of ^*^CO intermediates directly accelerates the C─C coupling process on Cu sites. As the rate‐determining step for the formation of C_2_ products, the enhanced reaction rate of C─C coupling ultimately drives the efficient generation of C_2_ products.

Bridge‐adsorbed CO (^*^CO_B_) intermediates were detected in the 1700–1800 cm^−1^ range, with their surface coverage following the same trend as linearly adsorbed ^*^CO_L_ across all four catalysts. While ^*^CO_B_ is generally considered relatively inert [50, 51, 52, 53], it can undergo hydrogenation to form ^*^CHO intermediates [54, 55] The ^*^CHO intermediate can further couple with ^*^CO_L_ to generate ^*^COCHO, a well‐documented key intermediate in C_2_ product formation [56] Subsequent reduction of ^*^COCHO enables the synthesis of high‐value multi‐carbon products such as ethanol and ethylene. The ^*^CHO intermediate was detected on both CuAg@MSN‐3 and CuAg/xc‐72, but its spectral intensity was significantly higher on CuAg@MSN‐3. Notably, no ^*^CHO signals were observed on Cu@MSN or Ag@MSN. These results indicate that high ^*^CO_B_ coverage favors the hydrogenation pathway to form ^*^CHO intermediates, underscoring the critical role of ^*^CO_B_ in C_2_ product formation.

A ^*^COCHO intermediate signal was observed at ∼1540 cm^−1^ for CuAg@MSN‐3 and CuAg/xc‐72 [57, 58]. Meanwhile, a peak corresponding to ^*^COCO was detected at ∼1560 cm^−1^ for Cu@MSN and CuAg/xc‐72 [59]. These results reveal that C─C coupling on CuAg@MSN‐3 proceeds primarily via the ^*^CO + ^*^CHO pathway, while Cu@MSN favors the ^*^CO + ^*^CO route. For CuAg/xc‐72, both pathways operate simultaneously. Although a peak appeared at 1580 cm^−1^ for Ag@MSN, it was assigned to the asymmetric O─C─O stretch of ^*^COO^−^ intermediates [60], since no C_2_ products were formed. Figure 4f summarizes the intensities of key C─C coupling intermediates. CuAg@MSN‐3 displays the strongest characteristic intensities, consistent with its superior C_2_ selectivity. In addition, a ^*^OC_2_H_5_ intermediate peak was identified at 1350 cm^−1^ for CuAg@MSN‐3 [61]. A peak at 1480 cm^−1^, assigned to CO_3_^2−^ from the electrolyte, appeared only for CuAg/xc‐72. This peak was absent for the MSN‐supported catalysts, likely due to the higher hydrophilicity of the xc‐72 support. On the basis of these results, a plausible catalytic mechanism for CuAg@MSN‐3 is proposed in Figure 4g. In CuAg@MSN‐3, Ag sites efficiently adsorb and activate CO_2_, rapidly generating CO. The mesoporous MSN framework confines these CO molecules, promoting their re‐adsorption on nearby Cu sites and establishing a high ^*^CO coverage. In this microenvironment, bridge‐adsorbed ^*^CO_B_ is protonated to ^*^CHO, which then couples with neighboring ^*^CO_L_ via a low‐energy‐barrier pathway to form ^*^COCHO. This kinetically favorable C─C coupling step directs the reaction selectively toward C_2_ products.

**FIGURE 5:**
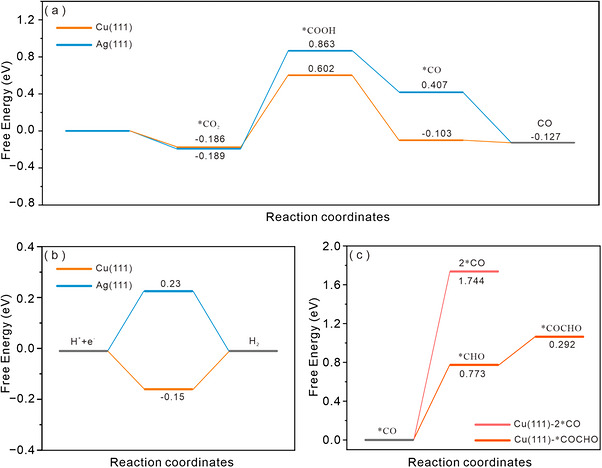
(a) The calculated Gibbs free energy diagram of CO_2_→^*^COOH→^*^CO→ CO on Cu(111) and Ag(111) catalysts. (b) The calculated Gibbs free energy diagram of H^*^ + H^*^ → H_2_ on Cu(111) and Ag(111) catalysts. (c) The calculated Gibbs free energy diagram of ^*^CO + ^*^CO^*^→COCO and the formation of ^*^CO + ^*^CHO→^*^COCHO on the Cu(111) surface.

DFT calculations were performed to gain mechanistic insight into the synergistic catalysis of CuAg@MSN. As illustrated in Figure 5a and Figures S25, S26, CO_2_ adsorption on both Cu(111) and Ag(111) surfaces is thermodynamically favorable, with similar adsorption free energies. However, the energy barrier for ^*^CO_2_ protonation to ^*^COOH is considerably lower on Cu(111) than on Ag(111), indicating that Cu sites are more active for the initial activation of CO_2_ toward ^*^CO formation. Notably, ^*^CO desorption to form gaseous CO shows a more negative free‑energy change on Ag(111) than on Cu(111). This thermodynamic driving force promotes CO spillover from Ag to Cu sites, providing a clear thermodynamic basis for the observed tandem catalytic behavior.

To quantitatively clarify the origin of CO vs. H_2_ selectivity, we computed the free‐energy profiles for the HER on Cu(111) and Ag(111) surfaces (Figure 5b). Cu(111) exhibits moderate ^*^H binding strength, which imparts appreciable HER activity. In contrast, Ag(111) binds ^*^H too weakly to support efficient HER, endowing Ag sites with inherently high CO selectivity under CO_2_ electroreduction conditions. Within the nanoporous MSN framework, the diffusion distance of CO molecules generated on Ag sites is significantly shortened. This enhances the probability of CO re‐adsorption on adjacent Cu sites, thereby increasing the surface coverage of ^*^CO on Cu.

Concerning the rate‑determining step for C_2_ production, in situ infrared measurements identified the ^*^CHO intermediate. We therefore examined the energetically most favorable ^*^CO‐^*^CHO coupling pathway (Figure 5c and Figure S27). Direct coupling of two ^*^CO to ^*^COCO shows an excessively high energy barrier of 1.74 eV, which severely hinders C_2_ generation. In contrast, protonation of ^*^CO to ^*^CHO followed by ^*^CO‐^*^CHO coupling to ^*^CHOCO proceeds with a much lower barrier of 1.06 eV, in good agreement with the experimentally observed ^*^CHO signal. Collectively, the catalytic mechanism of CuAg@MSN can be described as follows, Ag sites selectively convert CO_2_ to CO, which then spills over and re‑adsorbs onto neighboring Cu sites. The confinement effect of the porous MSN framework amplifies this process, promotes ^*^CO hydrogenation to ^*^CHO, and enables low‑barrier ^*^CO‐^*^CHO coupling to ^*^CHOCO. This favorable pathway ultimately leads to highly efficient and selective formation of C_2_ products.

### 3.4 MEA Performance

**FIGURE 6:**
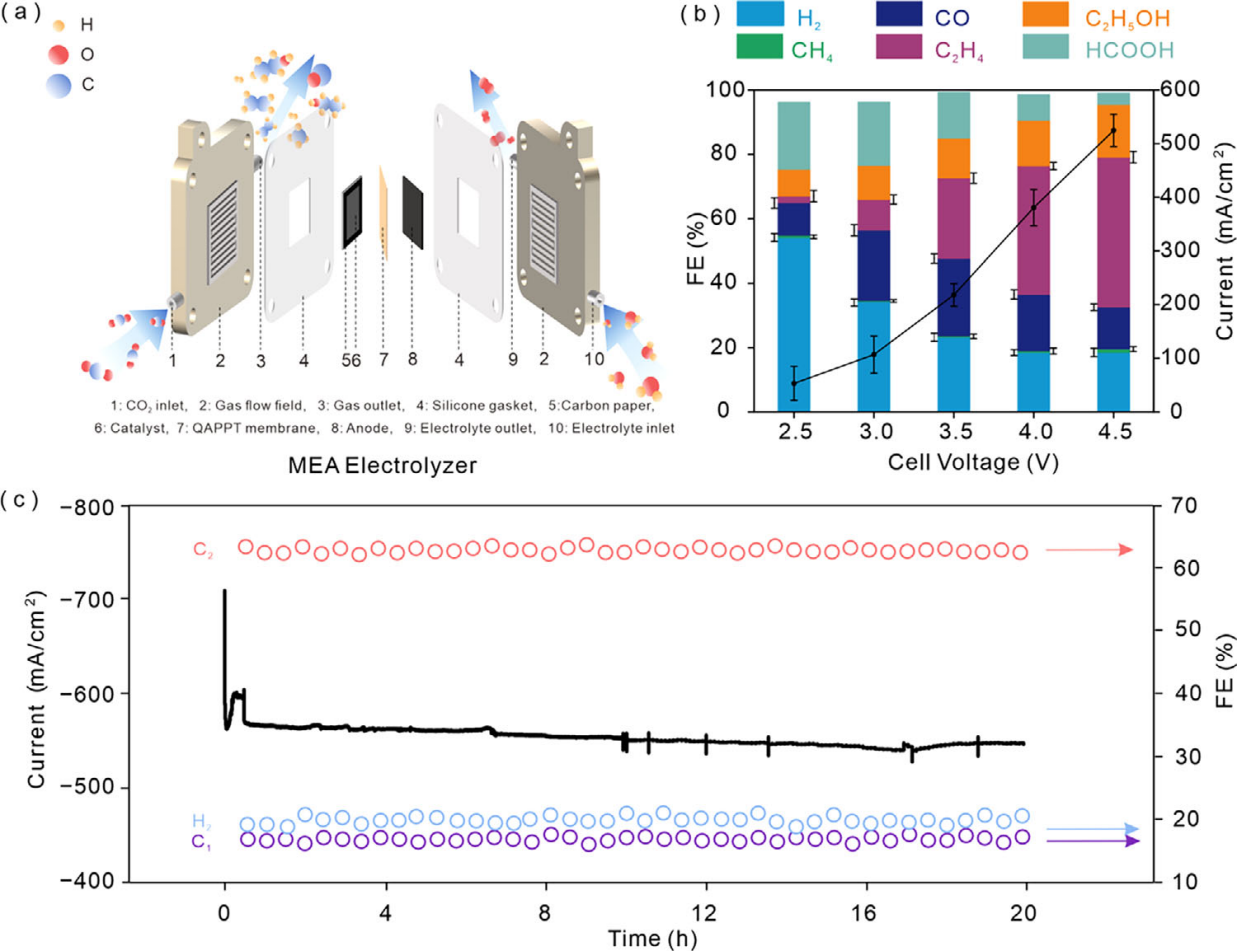
MEA electrolyser operated with 0.1m KOH aqueous solution. (a) Structural illustration of the MEA electrolyserb. (b) performance of the CO_2_RR. (c) Long‐term catalysis stability.

To address the low CO_2_ solubility and mass‑transport limitations in aqueous electrolytes, we assembled a zero‑gap electrolyzer using a gas‑diffusion electrode (GDE) integrated with a membrane‑electrode assembly (MEA). The CO_2_RR performance of CuAg@MSN‑3 was evaluated in 0.1 m KOH solution (Figure 6a). Figure 6b shows the product Faradaic efficiencies as a function of cell voltage. With increasing cell voltage, the H_2_ FE decreases continuously, indicating effective suppression of HER. Meanwhile, the FE of C_2_ products (primarily C_2_H_4_ and C_2_H_5_OH) increases steadily, reaching 62.9% at 4.5 V, with a total current density of 524.7 mA cm^−2^. The corresponding C_2_ partial current density is 330 mA cm^−2^, confirming that CuAg@MSN‑3 maintains excellent C─C coupling activity even under high‑current, industrially relevant conditions.

Long‑term durability was examined by continuous electrolysis at 4.5 V for 20 h (Figure 6c). Over the test period, the FEs of H_2_, C_1_, and C_2_ products varied by less than ± 2.4%, and the total current density decayed by less than 5%. No obvious salt precipitation, electrode flooding, or catalyst delamination was observed, demonstrating the excellent stability and robustness of the GDE‑MEA system based on CuAg@MSN‑3.

## 4 Conclusions

In summary, a series of CuAg@MSN tandem catalysts with tunable Cu/Ag ratios were successfully fabricated via a dual‐phase templating strategy combined with gas‐phase reduction, and their electrocatalytic CO_2_ reduction performance was systematically evaluated. Comprehensive structural and morphological characterizations confirm that metallic Cu and Ag nanoparticles are uniformly encapsulated within the mesoporous channels of the MSN framework, preserving the structural integrity of the support while ensuring effective dispersion of active sites. Operando infrared spectroscopy combined with DFT calculations elucidates the unique catalytic mechanism of CuAg@MSN, CO generated at Ag sites undergoes spillover and is confined within the mesoporous MSN channels, which facilitates its re‐adsorption on adjacent Cu sites and significantly enhances ^*^CO intermediate coverage on Cu surfaces. This coordinated regulation of reaction intermediates effectively promotes C─C coupling and boosts the selectivity toward multi‐carbon products. The optimized CuAg@MSN‐3 catalyst achieves a remarkable C_2_ Faradaic efficiency of 75.4% at −1.4 V vs. RHE, outperforming the Cu/xc‐72 counterpart and monometallic catalysts. Furthermore, the catalyst effectively suppresses the competing HER and maintains excellent structural stability and catalytic activity during long‐term electrolysis. Importantly, the catalyst also exhibits outstanding performance under industrially relevant high‐current conditions in a zero‐gap GDE‐MEA electrolyzer, demonstrating its practical application potential.

This work highlights the synergistic effects of spatial confinement from the MSN framework and tandem catalysis between Cu and Ag bimetallic sites in enhancing CO_2_ electroreduction performance. More importantly, it provides a versatile design paradigm for the rational construction of efficient catalysts for the sustainable production of high‐value fuels and chemicals from CO_2_.

## Author Contributions

**Jiaying Zhang**: Carried out the experiments and analyzed data. **Junjie Huang**: Assembled MEA electrolyzer and analyzed data. **Siying Zhang**, **Yanjia Cui**, **Ketong He**, **Zijian Peng**, **Huihui Jiang**, **Sirui Deng**, **Zhuoyao Chen**, **Caili Yang**:Collected and analyzed experimental data. **Chao Kong**: Provided DFT calculation. **Yibing Song**: Revised the manuscript. **Zhen Li**: Provided resources, analyzed data, and wrote the draft. **Gongwei Wang** and **Lin Zhuang**: Revised the manuscript.

## Conflicts of Interest

The authors declare no conflicts of interest.

## Supporting information




**Supporting File**: advs76068‐sup‐0001‐SuppMat.docx

## Data Availability

The data that supports the findings of this study are available in the supplementary material of this article.
