# Supplementary material for: Confinement‐Driven CO Spillover in CuAg@MSN Tandem Catalysts Boosts C2 Selectivity Toward Electrocatalytic CO2 Reduction
Source: Adv Sci (Weinh). 2026 Jun 11;13(50):e76068. doi: 10.1002/advs.76068 (PMC13337123; doi:10.1002/advs.76068)
Supplement: Supplementary file 1 [file ADVS-13-e76068-s001.docx]

**Supporting Information**

**Confinement-Driven CO Spillover in CuAg@MSN Tandem Catalysts Boosts C_2_ Selectivity toward Electrocatalytic CO_2_ Reduction**

Jiaying Zhang^1^, Junjie Huang^1^, Siying Zhang^1^, Yanjia Cui^1^, Chao Kong^4^, Zijian Peng^1^, Huihui Jiang^1^, Sirui Deng^1^, Zhuoyao Chen^1^, Caili Yang^1^, Ketong He^1^, Zhen Li^1,*^, Yibing Song^1, *^, Gongwei Wang^2,*^ and Lin Zhuang^2,3^

*^1^ College of Chemistry & Chemical Engineering and Key Laboratory for Preparation and Application of Ordered Structural Materials of Guangdong Province, Shantou University, Shantou 515063, China*

*^2^* *College of Chemistry and Molecular Sciences, Hubei Key Lab of Electrochemical Power Sources, Wuhan University, Wuhan 430072, China*

*^3^* *The Institute for Advanced Studies, Wuhan University, Wuhan 430072, China*

*^4^ College of Petroleum and Chemical Engineering, Longdong University, Qingyang 745000, China*

^*^Corresponding e-mails: [lizhenlicp@stu.edu.cn;](mailto:lizhenlicp@stu.edu.cn;) ybsong@@stu.edu.cn; gwwang@whu.edu.cn

**Experimental Section**

**Material characterizations**

The surface morphology was characterized by scanning electron microscopy (SEM) using a Gemini 300 field-emission scanning electron microscope. Transmission electron microscopy (TEM), high-resolution transmission electron microscopy (HRTEM), and energy-dispersive spectroscopy (EDS) elemental mapping were performed on a JEM-F200 Plus microscope operating at 200 kV. The crystalline phase was analyzed by X-ray diffraction (XRD) using a MiniFlex diffractometer with a scanning speed of 5°/min over the range of 10° to 80°. X-ray photoelectron spectroscopy (XPS) signals were collected using a Thermo Fisher ESCALAB 250 Xi spectrometer equipped with a monochromatic Al Kα X-ray source (1486.6 eV) at 12.0 kV and 6.0 mA. The Cu, Ag, and C spectra were fitted using Avantage software. The functional groups were identified by attenuated total reflectance Fourier-transform infrared spectroscopy (ATR-FTIR) using a Nicolet is 50 spectrometer over the spectral range of 4000 to 400 cm⁻¹.

**Electrochemical measurements**

Electrochemical tests were conducted using an electrochemical workstation (CHI660E, Shanghai CH Instrument Co., Ltd., China). A typical H-type electrolysis cell was employed for the electrolysis reaction at room temperature. A graphite rod was used as the counter electrode, a glassy carbon disk electrode with a diameter of 1 cm as the working electrode, and a saturated calomel electrode (SCE) as the reference electrode. A cation exchange membrane (Nafion-117) was used to separate the cathode and anode compartments, both of which were filled with 0.1 M KHCO_3_ electrolyte solution.To prepare the working electrode, 100 µL of ink (2 mg of catalyst dispersed in 1 mL of 0.05 wt% Nafion ethanol solution) was dropped onto the surface of the glassy carbon electrode and allowed to dry naturally before testing. Prior to electrochemical testing, high-purity CO_2_ gas (purity 99.999%) was bubbled through the electrolyte solution for at least 30 minutes to obtain a CO_2_-saturated 0.1 M KHCO_3_ solution (pH = 6.8). All potentials measured in this study were referenced to the reversible hydrogen electrode (RHE) using the following conversion formula: E (vs. RHE) = E' (vs. SCE) + 0.0591×pH + 0.241. All electrochemical measurements were carried out in a membrane electrode assembly (MEA) device at room temperature. The working electrode was AvCarbon GDS 3250 carbon paper (1.5 cm × 1.5 cm, Ballard), and the counter electrode was a titanium sheet (1.5 cm × 1.5 cm) loaded with 2 mg/cm^2^ of IrO_2_. The anode catalyst consisted of 90 wt% IrO_2_ and 10 wt% quaternized poly(phenylene oxide) (QAPPT), which was soaked overnight in 1 M KOH solution at 80 °C to complete ion exchange prior to testing. The cathode and anode chambers were separated by a quaternized QAPPT membrane. The electrolyte was 0.1 M KOH solution, continuously circulated through the system at a flow rate of 50 sccm. A catalyst ink was prepared by uniformly dispersing 4.5 mg of catalyst and 0.3 mg of QAPPT in a DMSO-ethanol mixture, which was then coated onto the carbon paper. During the tests, carbon dioxide gas was continuously bubbled into the cathode chamber at a constant flow rate of 30 sccm. Gaseous products were detected using an online gas chromatograph (GC) equipped with a thermal conductivity detector (TCD) and two flame ionization detectors (FID), specifically a Shimadzu GC-2014C model. Quantitative analysis of the gaseous products was performed using the external standard method, with standard curves prepared from gases of different concentrations. Liquid products generated from CO_2_RR were analyzed by ¹H NMR using a Bruker 400 MHz spectrometer. After testing, the sampled electrolyte was mixed with D_2_O and dimethyl sulfoxide (DMSO) standard solution in water suppression mode. Each data point represents the average value obtained from at least three independent GC or NMR analyses.

**ECSA measurements**

The double-layer capacitance (Cdl) has been widely used for the determination of the electrochemically active surface area (ECSA). The Cdl value is obtained by conducting cyclic voltammetry in the non-Faradaic region at different scan rates within the potential range of 0.00 V to 0.60 V versus the reversible hydrogen electrode (RHE). The current differences at 0.12 V are compared and fitted for various scan rates (20, 40, 60, 80, and 100 mV/s), and the Cdl value is then calculated using the equation Cdl = Ic/V. Here, Cdl represents the double-layer capacitance (μF), Ic is the charging current (μA), and V is the scan rate (V/s). In this study, polycrystalline copper was chosen as the reference benchmark for calculating the ECSA of various electrocatalysts, with a double-layer capacitance value of 29 μF/cm^2[1]^.

**In-situ ATR-SEIRAS measurements**

Prior to conducting the in situ attenuated total reflection surface-enhanced infrared absorption spectroscopy (ATR-SEIRAS) measurements, the ATR-Si prism was thoroughly cleaned. A thin gold film (Au film) was then deposited on its reflective surface using the chemical deposition method reported in the literature ^[2-4]^. After deposition, the Au film was rinsed with deionized water, dried, and its resistance was measured to ensure a value of less than 10 Ω.The electrocatalyst ink was prepared by mixing 5 mg of catalyst, 40 µL of 5 wt% Nafion solution, and 1 mL of anhydrous ethanol. The mixture was sonicated for 30 minutes to achieve uniform dispersion. Subsequently, 100 µL of the prepared catalyst ink was dropped onto the Au film surface and allowed to dry naturally at room temperature.The measurements were performed on a Thermo Fisher Nicolet is 50 Fourier-transform infrared (FT-IR) spectrometer equipped with a customized three-electrode electrochemical cell . The Si prism coated with the catalyst served as the working electrode, a saturated calomel electrode (SCE) as the reference electrode, and a platinum sheet as the counter electrode. The electrolyte used was a 0.5 M KHCO_3_ solution. The CHI660E electrochemical workstation was employed to control the testing process. Before the in situ ATR-SEIRAS measurements, the electrolyte solution was purged with high-purity CO_2_ gas for 30 minutes to achieve saturation, and the CO_2_ flow was maintained throughout the entire testing process. Spectra were collected at selected potentials with a resolution of 8 cm^-1^ and 44 scans. The spectra were presented in absorption mode, with positive peaks indicating signal enhancement and negative peaks representing signal attenuation.After the in situ ATR-SEIRAS measurements were completed, the Si prism coated with the catalyst was immersed in aqua regia until all the gold film and catalyst residues were completely removed.

**Density functional theory (DFT) calculations**

All calculations were performed within the framework of spin-polarized density functional theory, using the projector augmented plane-wave (PAW) method as implemented in the Vienna Ab Initio Simulation Package (VASP). The exchange-correlation potential was treated with the generalized gradient approximation (GGA) proposed by Perdew, Burke, and Ernzerhof (PBE), and the long-range van der Waals interactions were described using the DFT-D3 method. The plane-wave cutoff energy was set to 450 eV, and the energy convergence criterion for the iterative solution of the Kohn-Sham equations was 10^-6^ eV. All structures were fully relaxed until the forces on the atoms were less than 0.02 eV/Å. Data analysis and visualization were carried out using VASPKIT and VESTA. To avoid interlayer interactions, a vacuum spacing of 20 Å was applied perpendicular to the surface.

Here, differences in Gibbs free energy (ΔG) for intermediates defined as:

$$\text{Δ}\text{G}\text{=Δ}\text{E}\text{+Δ}\text{E}_{\text{ZPE}}\text{−}\text{T}\text{Δ}\text{S}$$

(1)

where ΔG is the total energy difference between the slab and respective terminations computed by DFT-PBE. ΔE_ZPE_ and TΔS denotes differences in zero-point energy and entropy between adsorbed states of reaction intermediates and gap phase, respectively.

The adsorption energy Eads is expressed as

|  |  |
| --- | --- |

where is the total energy of slab A model with B adsorption, is the energy of a A slab, and is that for a B molecule.

**Fig. S1. (a)** The SEM images of CuAg@MSN, **(b)** The TEM images of CuAg@MSN.

**Fig. S2.** **(a)** Nitrogen sorption and pore textural properties of MSN, **(b)** The Pore Volume and Pore Width of MSN.

**Table S1.** The BET surface areas, pore volumes and average pore diameter of MSN.

| Sample | BET surface  Areas (m^2^/g) | Pore volumes  (cm^3^/g) | Average pore  Diameter (nm) |
| --- | --- | --- | --- |
| MSN | 228.710 | 0.073 | 5.470 |

**Fig. S3.** The XRD patterns for MSN.

**Fig. S4.** The XRD patterns for CuAg@MSN-1, CuAg@MSN-2, CuAg@MSN-3, CuAg@MSN-4 and CuAg@MSN-5.

**Fig. S5.** The XPS spectra of Cu kinetic energy in **(a)** CuAg@MSN **(b)** CuAg/xc-72 **(c)** Cu@MSN electrocatalysts.

**Fig. S6.** The SEM image of **(a)** Cu@MSN **(b)** Ag@MSN **(c)** CuAg/xc-72.

**Fig. S7.** The TEM image of **(a)** Cu@MSN **(b)** Ag@MSN **(c)** CuAg/xc-72.

**Fig. S8.** HAADF-STEM of **(a)** CuAg@MSN **(b)** CuAg/xc-72 **(c)** Cu@MSN **(d)** Ag@MSN.

**Fig. S9.** EDS elemental mapping of **(a)** CuAg/xc-72 **(b)** Cu@MSN **(c)** Ag@MSN.

**Fig. S10.** Nitrogen sorption and pore textural properties of **(a)** CuAg@MSN-3 **(b)** CuAg/xc-72 **(c)** Cu@MSN **(d)** Ag@MSN.

**Fig. S11.** The Pore Volume and Pore Width of **(a)** CuAg@MSN-3 **(b)** CuAg/xc-72 **(c)** Cu@MSN **(d)** Ag@MSN.

**Table S2.** The BET surface areas, pore volumes and average pore diameter of catalysts.

| Sample | BET surface  Areas (m^2^/g) | Pore volumes  (cm^3^/g) | Average pore  Diameter (nm) |
| --- | --- | --- | --- |
| CuAg@MSN-3 | 188.507 | 0.063 | 7.961 |
| CuAg/xc-72 | 74.918 | 0.001 | 13.029 |
| Cu@MSN | 266.564 | 0.068 | 5.402 |
| Ag@MSN | 219.563 | 0.061 | 8.297 |

**Fig. S12.** The SEM images of MSN with the TMB dosage of **(a)** 1.65 mL, **(b)** 1.75 mL, **(c)** 1.85 mL, and **(d)** 1.95 mL during the synthesis process**.**

**Fig. S13.** CO_2_RR performance of CuAg@MSN catalysts (40% Cu/15% Ag) supported on MSNs with different pore sizes. (a-d) Electrocatalytic performance of catalysts with MSN pore sizes of 6.2 nm, 9.5 nm, 13.9 nm, and 15.2 nm, respectively. (e) Comparison of C_2_ product selectivity for the above catalysts at −1.4 V vs. RHE.

**Fig. S14.** CO_2_RR performance of CuAg@MSN catalysts with a fixed Ag loading of 15% and varied Cu loadings. (a–d) Catalytic performance of catalysts with 30% Cu, 35% Cu, 40% Cu, and 45% Cu, respectively. (e) Comparison of C₂ product selectivity for the above catalysts at −1.4 V vs. RHE.

**Fig. S15.** CO_2_RR performance of CuAg@MSN catalysts with a fixed Cu loading of 40% and varied Cu loadings. (a–e) Catalytic performance of catalysts with 5% Ag, 10% Ag, 15% Ag, 20% Ag, and 25% Ag, respectively. (f) Comparison of C₂ product selectivity for the above catalysts at −1.4 V vs. RHE.

**Fig. S16.** Current−voltage curves on CuAg@MSN-3, CuAg/xc-72, Cu@MSN and Ag@MSN catalysts obtained from the linear sweep voltammetry scans in **(a),** CO_2_ **(b),** N_2_. Scan rate: 20 mV s^−1^.

**Fig. S17.** Performance of the CO_2_RR catalyzed by **(a)** CuAg@MSN-3 **(b)** CuAg/xc-72 **(c)** Cu@MSN **(d)** Ag@MSN.

**Table S3.** The ECSA of electrocatalysts determined by measuring the double-layer capacitances.

| Electrocatalyst  quality  ( mg) | | Doublelayer  capacitance  ( μF) | ECSA  (cm2/ mg) |
| --- | --- | --- | --- |
| CuAg@ MSN-3 | 0.2 | 390 | 67 |
| CuAg/xc-72 | 0.2 | 544 | 94 |
| Cu@ MSN | 0.2 | 335 | 58 |
| Ag@ MSN | 0.2 | 85 | 15 |

**Fig. S18.** CV curves of **(a)** CuAg@MSN-3 **(c)** CuAg/xc-72 **(e)** Cu@MSN **(g)** Ag@MSN conducted in 0.1 M KHCO_3_ at different scan rates of 20, 40, 60, 80, and 100 mV s^-1^. The double-layer capacitances of **(b)** CuAg@MSN-3 **(d)** CuAg/xc-72 **(f)** Cu@MSN **(h)** Ag@MSN.

**Fig. S19.** Long-term catalysis stability of **(a)** CuAg/xc-72 **(b)** Cu@MSN **(c)** Ag@MSN.

**Fig. S20.** The SEM image of **(a)** CuAg@MSN-3 **(b)** CuAg/xc-72 **(c)** Cu@MSN **(d)** Ag@MSN after CO_2_RR.

**Fig. S21.** The TEM image and corresponding elemental mapping of Cu, C and Ag species of **(a)** CuAg@MSN-3 **(b)** CuAg/xc-72 **(c)** Cu@MSN **(d)** Ag@MSN after CO_2_RR.

**Fig. S22.** The XRD patterns of **(a)** CuAg@MSN-3 **(b)** CuAg/xc-72 **(c)** Cu@MSN **(d)** Ag@MSN after CO_2_RR.

**Table S4.** Quantitative XRD analysis coupled with Scherrer equation calculations.

| Sample | Before CO_2_ RR | | After CO_2_RR | |
| --- | --- | --- | --- | --- |
|  | Cu | Ag | Cu | Ag |
| CuAg@ MSN | 40.19 | 25.18 | 41.26 | 26.75 |
| CuAg/xc-72 | 25.33 | 28.00 | 37.37 | 29.24 |
| Cu@ MSN | 41.56 | ----- | 43.23 | ----- |
| Ag@ MSN | ----- | 26.77 | ----- | 27.38 |

**Fig. S23.** Tafel slope analysis of CuAg@MSN-3, CuAg/xc-72, Cu@MSN and Ag@MSN in **(a)** N_2_ and **(b)** CO_2_ saturated 0.1 M KHCO_3_ aqueous solution.

**Fig. S24.** ATR-FTIR spectra of **(a)** CuAg@MSN-3 **(b)** CuAg/xc-72 **(c)** Cu@MSN **(d)** Ag@MSN.

**Fig. S25.** Schematic representation of CO formation mechanism on Ag(111).

**Fig. S26.** Schematic representation of CO formation mechanism on Cu(111).

**Fig. S27.** Schematic representation of **a,** *COCO and **b,** *COCHO formation mechanism on Cu(111).

[1] Wei, X.; Yin, Z. L.; Lyu, K. J.; et al. Highly Selective Reduction of CO_2_ to C Hydrocarbons at Copper/Polyaniline Interfaces. *ACS Catal.* **2020**, 10 (7), 4103–4111.

[2] Yan, Y. G.; Li, X. X.; Huo, S. J.; et al. Ubiquitous Strategy for Probing ATR Surface-Enhanced Infrared Absorption at Platinum Group Metal-Electrolyte Interfaces. *J. Phys. Chem. B.* **2005**, 109 (16), 7900–7906.

[3] Chang, X. X.; Xiong, H. C.; Xu, Y. F.; et al. Determining Intrinsic Stark Tuning Rates of Adsorbed CO on Copper Surfaces. *Catal. Sci.* *Technol.* **2021**, 11 (20), 6825–6831.

[4] Yang, B. P.; Liu, K.; Li, H. J. W.; et al. Accelerating CO_2_ Electroreduction to Multicarbon Products via Synergistic Electric−Thermal−ield on Copper Nanoneedles. *J. Am. Chem. Soc.* **2022**, 144 (7), 3039−3049.
